# Lymphoscintigraphy is frequently recommended but seldom used in a “real world setting”

**DOI:** 10.1016/j.jvsv.2023.101738

**Published:** 2023-12-14

**Authors:** Tina Moon, Thomas F. O'Donnell, Derek Weycker, Mark Iafrati

**Affiliations:** a Department of Surgery, Tufts Medical Center, Boston, MA; b Division of Vascular Surgery, Cardiovascular Center, Tufts Medical Center, Boston, MA; c Policy Analysis Inc, Chestnut Hill, MA; d Department of Vascular Surgery, Vanderbilt University Medical Center, Nashville, TN

**Keywords:** Lymph nodes, Lymphatic disorders, Lymphedema, Lymphoscintigraphy

## Abstract

**Objective:**

Lymphedema (LED) lacks a standard, simple, guiding noninvasive diagnostic test, unlike the two other circulatory disorders—arterial or venous disease. Lymphoscintigraphy (LSG) has been recommended by several guidelines as the diagnostic test of choice for LED. Several recent expert panels, however, have suggested from anecdotal experience that LSG was used infrequently, and that the diagnosis of LED is usually based on clinical examination.

**Methods:**

To determine the use of LSG in a large real-world LED population, the International Business Machines MarketScan Research Database was examined from April 2012 to March 2020 for patients with a new diagnosis of LED (the index date). Use of LSG (LSG+) was ascertained during the period beginning 12 months prior to the initial coding of a LED diagnosis and ending 12 months after the index date based on the corresponding Current Procedural Terminology code; LSG use for sentinel node mapping at the time of oncologic surgery was excluded. Demographic profiles, comorbidities, and causes of LED among patients with and without evidence of LSG were characterized.

**Results:**

We identified 57,674 patients, aged ≥18 years, who had a new diagnosis of LED and health care coverage for ≥12 months before and after this index date. Only a small number (1429; 2.5%) of these patients underwent LSG during the study period. The LSG + cohort was younger (53.7 vs 60.7 years), had a higher proportion of women (91.3% vs 73.4%), but a lower percentage of diabetes (12.8% vs 27.5%), heart failure (2.2% vs 8.7%), hypertension (32.4% vs 51.0%), and obesity (15.1% vs 22.2%) compared with the LED population who did not undergo LSG (all *P* < .001). Most importantly, the use of LSG for diagnosis varied with the etiology of LED (LSG was most frequently utilized among patients with melanoma-LED (9.5%) and patients with breast cancer-LED (6.7%), in contrast to patients with advanced venous disease-related LED (1.1%; *P* < .05 for both comparisons).

**Conclusions:**

Despite four guidelines recommending LSG, including the Guidelines of the American Venous Forum (Handbook of Venous and Lymphatic Disease-4th edition), which recommended LSG “for the initial evaluation of patients with LED” with a 1B recommendation, LSG plays a minor role in establishing the diagnosis of LED in the United States. This underlines the need for a better, simple diagnostic test for LED to complement clinical examination.

## Graphical abstract

**Figure:**
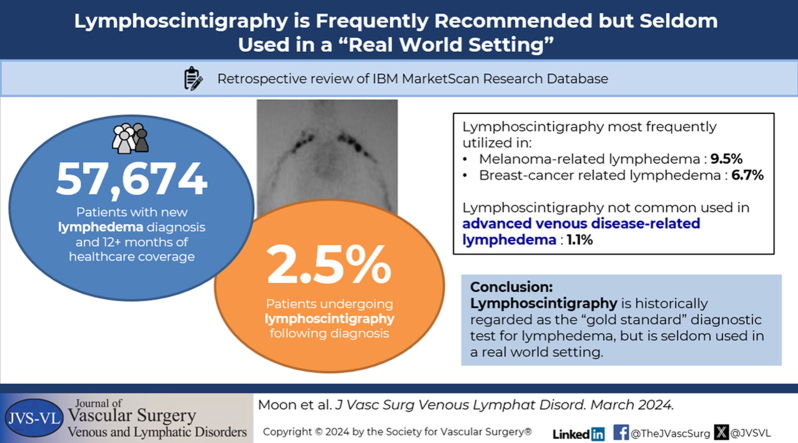

**Article Highlights**

• **Type of Research:** Retrospective observational cohort study
• **Key Findings:** Evaluation of 57,674 patients with a new diagnosis of lymphedema demonstrated that only 1429 (2.5%) underwent lymphoscintigraphy. Lymphoscintigraphy was most frequently utilized among patients with melanoma-related lymphedema (9.5%) and patients with breast cancer-related lymphedema (6.7%) in contrast to advanced venous disease-related lymphedema (1.1%).
• **Take Home Message:** Lymphoscintigraphy has historically been regarded as the “gold standard” diagnostic test of choice for lymphedema. Despite these recommendations, our results demonstrate that lymphoscintigraphy is seldom used in the real-world setting for the diagnosis of lymphedema.

Lymphedema (LED) is a chronic disease of the lymphatic system caused by the progressive accumulation of protein-rich fluid in the interstitium, ultimately leading to inflammation, fibrosis, and adipose tissue hypertrophy.^1^ The etiology of LED is categorized into primary and secondary LED. Primary LED is related to a genetic and/or congenital condition caused by a defect in the lymphatic system, whereas secondary LED is caused by damage to the lymphatic system from surgery, radiation, trauma, tumor burden, chronic venous insufficiency, infection, etc (Fig 1).^2^

**Fig 1:**
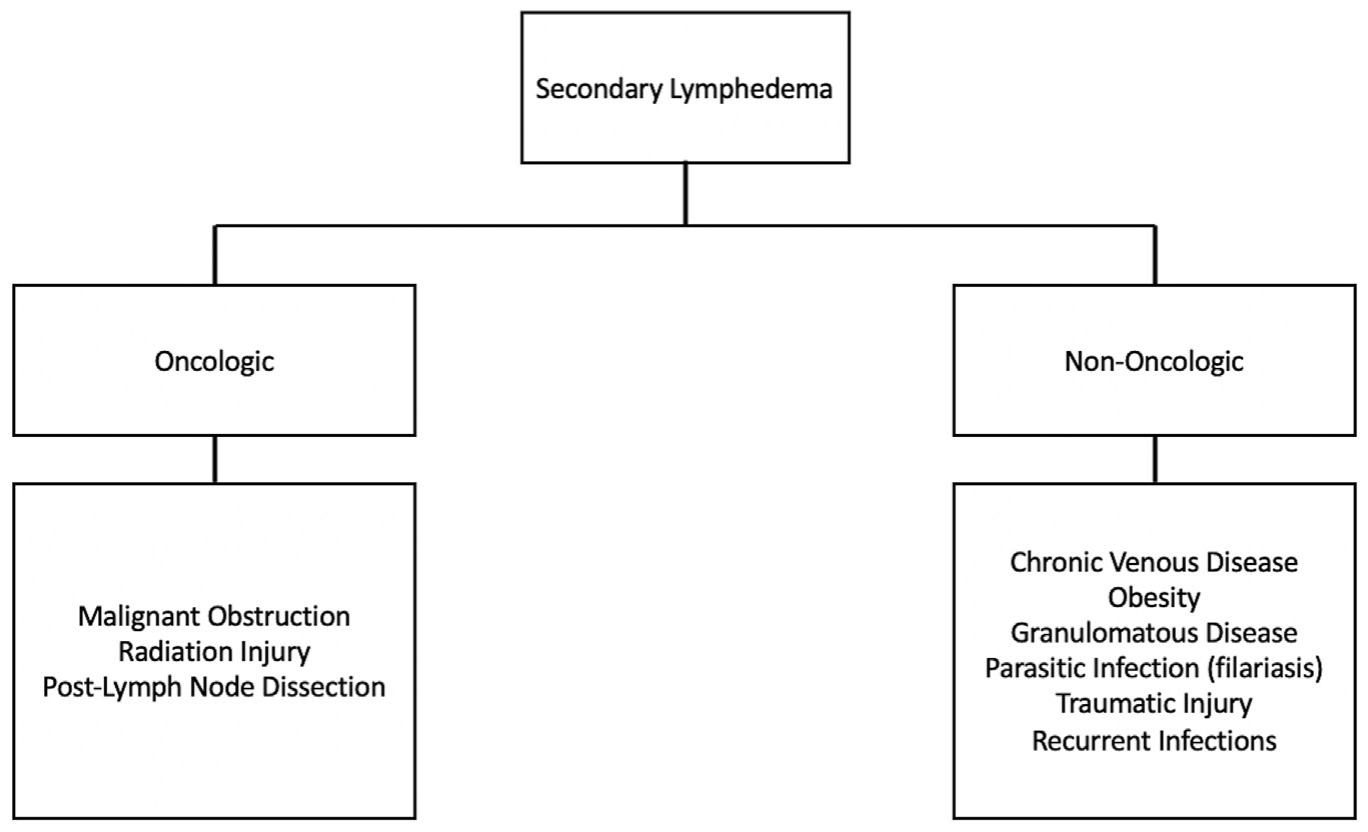
Types of secondary lymphedema (LED) identified in this data set.

Often heralded as the “forgotten vascular disease” due to its limited recognition in comparison to venous and arterial disease, LED can be frequently misdiagnosed until its late stages.^3^ Supplanting lymphangiography, lymphoscintigraphy (LSG) has historically been regarded as the gold standard diagnostic test for LED.^4^ During LSG, a small amount of radioactive colloid tracer is injected into the subcutaneous tissue. Sequential gamma camera images are then performed to capture the lymphatic system as the tracer migrates proximally to the nearest nodal basin.^5^ A recent systemic review of various LED clinical practice guidelines demonstrated that LSG was favored as the principal method for diagnosing patients with LED when the cause of swelling was unclear or to rule out LED as a part of the differential diagnosis.^6^

Although LSG is available at most medical centers in the United States (U.S.), there exists significant variability in injection types (intradermal vs subcutaneous), radiotracer types (sulfur colloid, albumin-nanocolloid, phytate, antimony sulfide), acquisition type (dynamic vs static), acquisition times, and physiologic state (at rest vs during stress activity).^7^ These differences across institutions lead to challenges in interpreting and comparing data. Additionally, the images produced with LSG (Fig 2.) are often of poor quality with very limited ability to assess anatomical details of the patient’s lymphatic system.^8^ Recent expert panels suggested from anecdotal experience that LSG was used infrequently, and the diagnosis of LED is usually based on clinical examination.

**Fig 2:**
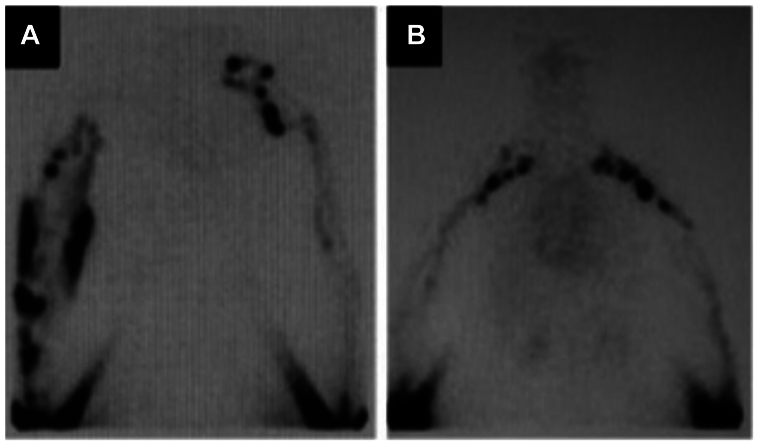
Sample lymphoscintigrams of the upper limbs of two breast cancer survivors with lymphedema (LED) of the right arms **(A** and **B)**. However, the lymphoscintigram in panel B appears normal. Images reproduced from Mihara, et al^10^ under the Creative Commons Attribution license.

The purpose of this study was to determine the proportion of patients with LED in a real-world setting who underwent LSG to support this diagnosis, and to determine any differences in demographic profile, clinical characteristics, comorbidities, or etiology of LED that might influence the decision to pursue LSG imaging.

## Methods

### Study design and data source

A retrospective observational cohort study was pursued using data from an integrated U.S. health care claims repository—the International Business Machines (IBM) MarketScan Commercial Claims and Encounters (CCAE) and Medicare Supplemental and Coordination of Benefits (MDCR) Databases. For this study, data spanned the period April 2012 through March 2020.

The CCAE Database includes health care claims and enrollment information from employer-sponsored plans throughout the U.S. that provide health benefits to working persons aged <65 years annually, including the employees, their spouses, and their dependents. The MDCR Database includes healthcare claims and enrollment information for retirees who are Medicare-eligible and have elected to enroll in employer-sponsored Medicare supplemental plans (and for which both the Medicare-paid amounts and employer-paid amounts are available).

Health care claims include medical (ie, facility and professional service) and outpatient pharmacy claims. Data available for each facility and professional service claim include the dates and places of service, diagnoses, procedures performed/services rendered, and quantity of services (professional service claims). Medical and pharmacy claims also include amounts paid (ie, reimbursed) by health plans and patients to health care providers for services rendered. Selected demographic and eligibility information is also available. Patient-level data can be arrayed chronologically to provide a detailed longitudinal profile of all medical and pharmacy services used by each plan member. The data set is deidentified with no patient identifying data provided to the investigators; therefore, Institutional Review Board Approval was not required.

### Study population

The study population comprised all patients aged ≥18 years who, between April 1, 2013, and March 31, 2019, had evidence of primary or secondary LED based on ≥1 International Classification of Diseases (ICD) diagnosis code for LED (ICD-9: 457.0, 457.1, 757.0; ICD-10: I97.2, I89.0, Q82.0) in the acute-care hospital (inpatient) setting, or ≥2 diagnosis codes for LED—at least 7 days apart—in the ambulatory (outpatient) setting. For all such patients, the date of the earliest diagnosis of LED was designated the “index date.” Because there is no current ICD-9 or ICD-10 code for phlebolymphedema, venous disease was identified by codes for: chronic venous insufficiency (n = 4132), venous leg ulcers (n = 4203), and postphlebitic syndrome (n = 71). We have used this method of ascertainment in other health care claims analyses of a different health care claims database.^3^

Patients were excluded from the study population if they had: ≥1 diagnosis code for LED (irrespective of care setting) at any time prior to their index dates; <12 months of health care coverage prior to their index dates (for purposes of characterizing LSG use as well as LED etiology, LED-associated conditions, and comorbidity profiles); <12 months of health care coverage following their index dates (for purposes of characterizing LSG use); and ≥1 diagnosis of head/neck cancer at any time preceding their index dates (to focus on peripheral LED).

### Study measures

Use of LSG for diagnostic purposes was ascertained during the period beginning 12 months prior to the initial coding of a LED diagnosis and ending 12 months thereafter based on the corresponding Current Procedural Terminology (CPT) code (78195: gamma-emitting radioactive tracer to diagnose and localize diseases of the lymphatics and lymph nodes). Use of CPT code 38792 (injection procedure for identification of sentinel node) was specifically rejected to avoid including patients who underwent sentinel node imaging for surgical purposes and not for establishing the diagnosis of LED. This focus was further strengthened by eliminating patients who had LSG to identify a sentinel node within 24 hours of a surgical procedure for cancer treatment.

### Baseline characteristics

Baseline characteristics of patients in the study population were ascertained during the 12-month period prior to their index dates, and included: demographic profile (age, sex, geographic region of residence, insurance type); clinical profile (LED-related conditions, comorbidities); and treatment profile (diuretics, anti-inflammatory agents). LED-related conditions and comorbidities were identified based on ≥1 inpatient encounter or ≥2 outpatient encounters with a corresponding diagnosis code.

### Data analysis

Baseline characteristics of patients in the study population, stratified by presence (vs absence) of undergoing LSG, were described. Categorical variables were reported as counts and percentages; for continuous variables, means and standard deviations were reported. Differences in baseline characteristics and LSG use between patients with LED with and without LSG were evaluated using an independent-samples *t*-test for continuous measures and χ^2^ test for categorical measures.

## Results

From April 2012 through March 2020, a total of 120,940 patients aged ≥18 years had a new diagnosis of LED based on the aforementioned criteria. Among these patients, 88,705 had ≥12 months of health care coverage preceding their index date, and 85,601 also had no evidence of head/neck cancer at any time preceding their index date. Of this cohort, 57,674 patients had ≥12 months of health care following their index date and thus qualified for inclusion in the study population. Among these patients, 1429 patients (2.5%) underwent LSG.

Compared with the LED population who did not undergo LSG (LSG −), the LED cohort who did undergo LSG (LSG +) was younger (53.7 vs 60.7 years; *P* < .001) and had a higher proportion of women (91.3% vs 73.4%; *P* < .001). Further subgroup analysis of associated comorbidities in the LSG + group (Table I) demonstrated a two-fold lower percentage of diabetes, three-fold lower proportion of heart failure, as well as a lower prevalence of hypertension and obesity compared with the LSG − group.

**Table I:** Complications, comorbid conditions, and prescribed medications for patients with lymphedema (*LED*)

	LSG + (n = 1429), %	LSG − (n = 56,245), %	*P* value
LED-associated conditions			
Iliac vein disorders (May-Thurner syndrome)	0.8	0.4	.0197
Cellulitis	6.3	16.0	<.0001
Lymphangitis	0.0	0.2	.1124
DVT	3.1	6.3	<.0001
Other infections	30.7	45.4	<.0001
Comorbidity profile			
Depression	30.7	28.1	.0268
Diabetes	12.8	27.5	<.0001
Heart failure	2.2	8.7	<.0001
With beta blocker therapy	1.5	5.6	<.0001
Without beta blocker therapy	0.6	3.0	<.0001
Hypertension	32.4	51.0	<.0001
Obesity	15.1	22.2	<.0001
Pulmonary hypertension	0.7	1.9	.0013
Renal disease	3.6	13.4	<.0001
Use of selected drugs/procedures			
Diuretics	22.0	40.6	<.0001
Dressings for venous leg ulcers	1.4	2.4	.0185

*DVT*, Deep venous thrombosis.

Cellulitis was three-fold lower in the LSG + group, whereas a history of deep venous thrombosis was two-fold lower. Diuretics were prescribed twice as frequently in the LSG – group, as were wound dressings. Use of LSG for diagnosis varied with the etiology of LED (Table II). LSG was most frequently utilized among patients with melanoma-LED (9.5%) and patients with breast cancer-LED (6.7%), in contrast to patients with advanced venous disease-related LED (1.1%; *P* < .05 for both comparisons).

**Table II:** Lymphoscintigraphy (*LSG*) use by etiology

LED etiology	Patients with LED (n = 57,674)	% LSG
Malignancy		
Breast cancer	12,580	6.7
Gynecologic cancer	838	1.3
Melanoma	455	9.5
Soft tissue sarcoma	136	1.5
Urologic cancer	656	0.5
Other cancer	495	1.0
Other		
Morbid obesity	3651	0.7
CVI/LVU/PPS	5609	1.1
Primary	35	0.0
Other cause	252	0.8
Multiple causes	2629	3.7
None (no evidence of aforementioned etiology)	27,338	0.8
Total	57,674	2.5

*CVI*, Chronic venous insufficiency; *LED*, lymphedema; *LVU*, leg venous ulcer; *PPS*, post-phlebitic syndrome.

To identify the prevalence of other alternative imaging studies for diagnosing LED, we assessed the proportion of tests performed within 1 year of the index date for the diagnosis of LED as well as 1 year after this date. Given the limitations of coding, one is unable to state that the tests were employed specifically for the diagnosis of LED. The most frequently performed study was duplex ultrasound, which was carried out in 50% of all patients. The frequency of computed tomography (CT) scans of the lower extremity was comparable to that of magnetic resonance imaging (MRI) studies of the lower extremity (4.1% and 4.4%, respectively), whereas these imaging studies were less frequently used in the upper extremity, around 1%. Lymphangiography was employed as a diagnostic study overall in only 17 patients (0.03%), of which nine patients had breast cancer-related LED (BCRL). Duplex scan was used in 26% of patients with BCRL but was employed in nearly 80% patients with venous-related LED. Despite BCRL affecting the upper extremities, CT scans or MRI imaging were used in less than 1%. The highest use of CT scans was in patients with primary LED where it was employed 15% of the time.

## Discussion

Our analysis of 57,674 patients with LED in a large deidentified health care insurance database demonstrates LSG is used infrequently for the diagnosis of LED, despite guideline recommendations as the diagnostic test of choice. Optimal management of LED requires a timely and accurate diagnosis to provide relief of the symptoms of heaviness and aching as well as reducing the risk of infection. Moreover, earlier treatment can reduce the permanent complication of subcutaneous fibrosis and scarring. Primary LED currently affects approximately 1 in 100,000 individuals in the U.S., whereas secondary LED is more common and affects approximately 1 in 1000 individuals.^9^ Diagnosis of LED in its early stages can be particularly challenging due to the slow progression of edema and is often not detected until patients have already suffered significant morbidity.^10^

LSG, which replaced the more invasive technique of lymphangiography, was first introduced by Sherman and Ter-Pogossian in 1953 and has since been considered the “gold standard” for the diagnosis of LED.^11^, ^12^, ^13^, ^14^, ^15^, ^16^, ^17^, ^18^, ^19^, ^20^ Despite the recommendations of several guidelines for LED, our real-world results demonstrate that only a minor proportion of patients with LED in this health care insurance data base undergo LSG. The LSG examination is dependent on the transport of a radiolabeled substance by the lymphatic system, which results in: (1) a semiquantitative measurement of the appearance time of the radionuclide, and (2) the pattern of distribution on imaging.^16^ Patel et al termed the visual or qualitative interpretation of LSG as detecting the presence of and defining the caliber of lymphatic vessels, lymph nodes, collateral networks, and delay in radionuclide uptake.^18^ By contrast, quantitative LSG involves objective measurement of radionuclide transit times, as determined by the uptake and clearance time from the site of injection and clearance time from the limb.

Cambria’s landmark 1993 prospective study on the value of LSG (Tc 99m-labeled antimony trisulfide colloid) was carried out in 188 patients (386 extremities), of whom 90% had lower extremity swelling.^19^ After initiating an exercise protocol, patients were imaged to determine the transport time to regional nodes. Seventy-nine extremities demonstrated a normal LSG pattern, whereas 125 patients with LED demonstrated a prolonged transport index (2.6 ± 0.5 vs 23.8 ± 1.5, respectively; normal TI < 5). In this study, LED was characterized based on clinical history and diagnostic examinations. Of 41 patients with abnormal venous studies, 18 (44%) had abnormal transport indices, which is indicative of concomitant lymphatic dysfunction. LSG excluded LED in one-third of their patients.

One of the main drawbacks of LSG is the lack of standardized protocols, making comparisons among the various studies difficult. Other disadvantages include low image resolution, invasiveness, radiation exposure, and inability to perform real-time imaging.^7^^,^^10^ An additional problem with LSG is the requirement for sequential imaging up to 3 hours in some protocols. Furthermore, successful LSG also depends on strong collaboration between nuclear medicine physicians and vascular specialists, which is highly variable across institutions. A recent consensus document of experts via the Delphi method showed a low agreement (42%) that “radionuclide lymphoscintigraphy should be recommended for patients with suspected or diagnosed lymphedema,” which is below the 70% threshold needed for consensus.^20^ The remaining 58% of panelists disagreed with the statement, and 7% strongly disagreed.

Various imaging modalities employing newly developed techniques are increasingly utilized for the diagnosis of LED, such as indocyanine green (ICG) lymphography, MRI lymphangiography, CT lymphangiography, photoacoustic lymphangiography, ultrasound, and near-infrared fluorescence lymphatic imaging (NIRFLI).^16^^,^^17^ Except for ultrasound, these modalities are more expensive on a per test basis, capital intensive, and are not generally a point-of-service test. A comparison of four diagnostic modalities (MRI, CT, LSG, and ICG lymphography) by Mihara et al demonstrated that ICG lymphography and MRI were superior to LSG for the diagnosis of secondary LED.^10^ A downside to ICG lymphography is the inability to detect lymphatic channels greater than 2 centimeters from the skin surface, which stands in contrast to NIRFLI, where the lymphatic vessels and their pumping function can be ascertained.^21^, ^22^, ^23^ A major disadvantage to MRI lymphangiography is the potential venous uptake of gadolinium-based contrast, which can lead to contaminated images.^22^^,^^24^ Furthermore, MRI lymphangiography is a significantly more costly imaging modality compared to ICG lymphography.^25^

Determining how frequently a diagnostic test is used can be difficult and encumbered by the bias of the study design. Studies employed to assess the accuracy of LSG cannot realistically portray the number of times that LSG is employed by their design, which lack a recognized “gold standard” for determining the presence or absence of LED.^19^ By contrast, clinical series on LED can report the number of times that LSG was employed. For example, Hassanein and colleagues reported on their series of 227 patients of whom 169 had clinically suspected LED.^15^ LSG confirmed LED in 162 (96%), whereas in 58 patients, by clinical criteria, a condition other than LED was posited—all had negative LSGs. One might also conclude conversely from this study that clinical diagnosis is quite sensitive and specific. One outcome of this study is that false negatives do occur, particularly in patients with primary LED—clinical evidence of lymphoedema but normal LSG. Finally, LSG may not be used to diagnose LED at all in clinical series, as in the recent 440-patient case series of Dean and associates, where the diagnosis and cause of LED was established on history and physical examination alone.^26^ A survey method has also portrayed the use of diagnostic tests for LED. A postal questionnaire was sent to all members of The Vascular Society of Great Britain and Ireland, which demonstrated that duplex ultrasound was employed by surgeons greater than 80% of the time and LSG 58% of the time for the initial evaluation of LED. In contrast, for the confirmation of LED, LSG was favored by a similar proportion, but 28% to 40% of respondents diagnosed LED by exclusion.^27^

“Big data,” such as deidentified health care insurance claims, provide a large reservoir of “real-world” patients that has been traditionally mined for medical resource utilization and costs. The prevalence of a disease can also be derived in the respective insured population.^3^^,^^28^ Big data is also an ideal data source for determining the utilization of a diagnostic test or procedure. Because these tests usually have a specific diagnostic code, the frequency and the reason for the test is available. Their study showed significant variability in the use of these two tests, which was related to the cause of cancer.

Our results demonstrate that LSG was largely employed in patients with BCRL. These findings correlate with the higher proportion of women (91.3% vs 73.4%) and younger age (53.7 vs 60.7 years) seen in the LSG + cohort. In our data we found that, compared with the advanced venous disease-related LED, patients with BCRL have a significantly lower proportion of comorbid conditions such as diabetes, heart failure, hypertension, and obesity. Furthermore, patients with BCRL also have a decreased usage of diuretic prescriptions and wound dressings. The higher proportion of patients with BCRL in the LSG + group also likely explains the lower incidence of cellulitis (4% vs 35.6%) and deep vein thrombosis (2.7% vs 12%) in this cohort. Cellulitis is less frequently seen in patients with upper extremity LED compared with lower extremity LED due to the associated perineal soiling and bacterial seeding that is seen in lower extremity LED. Advanced chronic venous disease is indeed probably more common than breast cancer as an etiology of secondary LED, but this etiology is under-recognized and, most importantly, under-reported. The preponderance of patients with secondary LED due to advanced chronic venous insufficiency Clinical, Etiology, Anatomic, Pathophysiology (CEAP) classification C3 through C6 have lymphatic involvement as earlier LSG studies and NIRFLI have demonstrated.^23^^,^^29^^,^^30^ Thus, with edema, one can suspect lymphatic involvement.

Current treatment for LED initially involves complete decongestive therapy, which includes some combination of manual lymphatic drainage, compression garments, physical therapy, pneumatic compression by either simple pneumatic compression device, advanced pneumatic compression device, and meticulous skin care.^31^ Although some practitioners consider imaging studies while developing treatment plans, there is no consensus on how to how to modify therapy based on LSG.^20^ However, therapists have recently utilized the dynamic real-time imaging possible with ICG to watch lymphatic transit during manual lymphatic drainage maneuvers, during their first treatment session.^32^ Utilization of this technology offers the potential to individualize and optimize therapy.

LSG depends upon functional criteria—delayed transit time of the radionuclide to the regional lymph nodes, as well as morphological features such as dermal backflow, asymmetric node uptake, and/or formation of collateral lymphatic channels to define “lymphatic drainage.” This diagnostic test may not be sensitive enough to depict important pathophysiologic changes, such as reduced contractility in the lymphangion and valvular dysfunction. Bollinger et al used fluorescence microlymphography to show the obliteration of parts of the lymphatic superficial capillary network and increased lymphatic capillary permeability.^33^ These changes are similar to those microscopic findings in the venous capillaries observed by Burnand and Browse in advanced chronic venous insufficency.^34^ In Akita et al’s comparative study of ICG vs LSG in 169 extremities with LED secondary to lymph node dissection and 65 extremities with primary LED, earlier and less severe dysfunction was found to be established by ICG for secondary LED.^35^ Furthermore, an important study by Jayaraj et al compared quantitative diagnostic criteria with several qualitative criteria, and found that 16 of 21 limbs (76%) with edema but a normal LSG had venous obstruction.^36^

Several surgical treatments are increasingly utilized for both the prophylactic prevention and/or treatment of LED, including lympho-venous bypasses, vascularized lymph node transplantation, suction-assisted protein lipectomy, etc.^21^ Preoperative planning for these lymphatic microsurgery procedures requires high quality imaging for the accurate depiction of anatomical detail and identification of targets for microvascular anastomoses.^24^ With its inability to visualize small lymphatic channels, LSG is an ineffective test for surgical planning. ICG lymphography with NIRFLI, however, provides real-time and highly detailed visualization of lymphatic flow.^16^^,^^37^

Our study is not without limitations. Our results are based on a retrospective review of a health insurance claims database and are therefore subject to the accuracy of this database. The identification of patients with LED and patients who underwent LSG is dependent on ICD-9 and ICD-10 codes, and these patients can only be identified if proper coding was performed. No LED etiology was found for 47.4% of patients, which is likely secondary to physicians not providing a defined etiology of the LED. Additionally, the IBM Market Scan database includes commercially insured patients and is limited to retirees who are Medicare-eligible and have elected to enroll in employer-sponsored Medicare supplemental plans (and for which both the Medicare-paid amounts and employer-paid amounts are available). This may introduce bias and reduce the overall generalizability of our results.

Lastly, the majority of patients had secondary LED, and only a small number were coded as primary LED (0.07%). This number was certainly limited by the exclusion criteria of patients <18 years of age, a time when the diagnosis of primary LED is usually made. The original group of patients was larger, and the study group was restricted to patients aged >18, those with health care coverage for 1 year preceding the index diagnosis of LED, and non-head and neck LED. Younger patients typically have their first instance of LED at the age of 14 to 15 (lymphedema praecox), which is the most common type of primary LED. The senior author has found LSG particularly useful in this situation. The “root causes” of secondary LED vary, and the treatment of the underlying cause will also. Phlebolymphedema results from venous hypertension with associated enhanced fluid filtration from the capillaries into the interstitial space. The reduction of the interstitial fluid cannot be matched by lymphatic transport, thus edema ensures (CEAP C3). More recent studies have focused on the lymphangion as the key unit of the lymphatics, and NIRFLI showed reduced contractions and valve dysfunction within this structures in limbs with phlebolymphedema.^23^ Recent analyses have demonstrated a need for continued LED-specific therapy (either manual lymphatic drainage or pneumatic compression) directed at lymphatic dysfunction in at least 50% of patients, because the edema does not reduce satisfactorily.^38^ Our findings are also compatible with the earlier important study by Raju et al, which found that diagnostic deficiencies are likely secondary to the misdiagnosis of venous LED as primary LED.^39^

## Conclusions

Despite multiple guidelines recommending the use of LSG, including the most recent guidelines of the American Venous Forum, our study shows that LSG plays a minor role in establishing the diagnosis of LED in the United States.^40^ This underlines the need for a better, more informative, and a simpler diagnostic test for LED to complement clinical examination and to guide therapy. Currently several modalities utilizing ultrasound, MRI, ICG, and CT have shown promise, but the role of each of these techniques in the diagnosis and management of LED remains to be established.

## Author Contributions

Conception and design: TT, TO, MI

Analysis and interpretation: TT, TO, DW, MI

Data collection: TO, DW

Writing the article: TT, TO, DW, MI

Critical revision of the article: TT, TO, DW, MI

Final approval of the article: TT, TO, DW, MI

Statistical analysis: DW

Obtained funding: Not applicable

Overall responsibility: MI

## Disclosure

D.W. received consultative reimbursement from Tactile for his independent performance of the health economic analysis. T.O. is a consultant to Tactile Medical.
